# PIK3CA Mutations in Advanced Cancers: Characteristics and Outcomes

**DOI:** 10.18632/oncotarget.716

**Published:** 2012-11-30

**Authors:** Filip Janku, Jennifer J. Wheler, Aung Naing, Vanda M. Stepanek, Gerald S. Falchook, Siqing Fu, Ignacio Garrido-Laguna, Apostolia M. Tsimberidou, Sarina A. Piha-Paul, Stacy L. Moulder, J. Jack Lee, Rajyalakshmi Luthra, David S. Hong, Razelle Kurzrock

**Affiliations:** ^1^ Department of Investigational Cancer Therapeutics (Phase I Clinical Trials Program), The University of Texas MD Anderson Cancer Center, Houston, Texas, USA; ^2^ Department of Biostatistics, The University of Texas MD Anderson Cancer Center, Houston, Texas, USA; ^3^ Molecular Diagnostic Laboratory, The University of Texas MD Anderson Cancer Center, Houston, Texas, USA; ^4^ Moores Cancer Center, The University of California San Diego, La Jolla, California, USA

**Keywords:** PIK3CA mutation, phenotypic taxonomy, clinical outcome

## Abstract

*PIK3CA* mutations are frequently diagnosed in diverse cancers and may predict response to PI3K/AKT/mTOR inhibitors. It remains unclear whether they are associated with other characteristics. We analyzed characteristics and outcome of 90 consecutive patients with diverse advanced tumors and *PIK3CA* mutations and 180 wild-type *PIK3CA* controls matched by tumor type, gender, and age referred to the Clinical Center for Targeted Therapy. *PIK3CA* and MAPK mutations (*KRAS*, *NRAS*, and *BRAF*) were analyzed using polymerase chain reaction-based DNA sequencing. The most frequent *PIK3CA* mutations were E545K (31/90, 34%), E542K (16/90, 18%) in exon 9, and H1047R (20/90, 22%) in exon 20. *PIK3CA* mutations compared to wild-type *PIK3CA* were associated with simultaneous *KRAS* (p=0.047) and MAPK mutations (p=0.03), but only MAPK mutations were confirmed as having an independent association in multivariate analysis. Rates of lung, bone, liver and brain metastases were similar in *PIK3CA*-mutant and wild-type patients. Patients with *PIK3CA* mutations treated on trials with PI3K/AKT/mTOR inhibitors had a higher partial/complete response (PR/CR) rate than wild-type *PIK3CA* patients treated with their best phase I therapy (10/56, 18% vs. 12/152, 8%; p=0.045), but not a prolonged progression-free survival. Patients with H1047R *PIK3CA* mutations had a higher PR/CR rate with PI3K/AKT/mTOR inhibitors compared to wild-type *PIK3CA* patients treated with their best phase I therapy (6/16, 38% vs. 12/152, 8%; p=0.003). In conclusion, *PIK3CA* mutations in diverse cancers were not associated with clinical characteristics, but were correlated with MAPK mutations. *PIK3CA* mutations, especially, H1047R, were associated with attaining a PR/CR to PI3K/AKT/mTOR pathway inhibitors.

## INTRODUCTION

*PIK3CA* mutations frequently occur in diverse cancers and are associated with constitutive activation of the PI3K/AKT/mTOR pathway.[1-5] In addition, *PIK3CA* mutations predicted sensitivity to PI3K/AKT/mTOR inhibitors in multiple tumor types in preclinical and early clinical experiments.[1, 2, 5-12] A seminal question is whether *PIK3CA* mutations are associated with a distinct phenotypic taxonomy. Retrospective studies in colorectal cancer demonstrated that *PIK3CA* mutations in exon 20 encoding for the kinase domain, but not in exon 9 encoding for the helical domain, are associated with resistance to EGFR-targeting monoclonal antibodies.[13] In addition, our group reported that, regardless of histology, *PIK3CA* mutations often coexist with mitogen-activated protein kinase (MAPK) mutations, such as mutated *KRAS*, *NRAS*, and *BRAF*.[14] A partial answer to the question posed about the relationship between *PIK3CA* mutations and specific subtypes of cancer is generally that different cancers seem to have different types of *PIK3CA* mutations and associations with still other mutations.[15] For example, in colorectal cancer *PIK3CA* mutations in exon 9, but not exon 20, trended toward an association with *KRAS* mutations, whereas only *PIK3CA* exon 20 mutations were associated with *KRAS* mutations in ovarian cancer.[13, 16] Other oncogenic mutations have also been correlated with clinical characteristics and outcome. For example patients with advanced cancers and *BRAF* mutations have less soft tissue, retroperitoneal, lung metastases and more brain metastases.[17] In colorectal cancer, *BRAF* mutations predicted poor outcome and *KRAS* mutations were associated with lung metastases.[13, 18] We investigated characteristics and outcomes of patients with advanced cancers with and without *PIK3CA* mutations.

## METHODS

### Patients

We retrospectively reviewed clinical and pathological characteristics and treatment outcomes of 90 consecutive patients with advanced tumors harboring *PIK3CA* mutations who had been referred to the Clinical Center for Targeted Therapy at The University of Texas MD Anderson Cancer Center (MD Anderson) starting in October 2008. To define distinguishing features of advanced cancers with *PIK3CA* mutations, we selected a control group of 180 patients with wild-type (wt) *PIK3CA* advanced cancers matched in a 2:1 ratio by tumor type, gender, and age (+/− 5 years) to patients with *PIK3CA* mutations referred to the MD Anderson Clinical Center for Targeted Therapy (CCTT) during the same period of time.

Data were collected from transcribed notes and radiology reports in the electronic medical record and other source documentation. Registering patients in the database, pathology assessment, and mutation analysis were performed at MD Anderson. The study and all treatments were conducted in accordance with the guidelines of the MD Anderson Institutional Review Board.

### Tissue samples and mutation analyses

*PIK3CA*, *KRAS*, *NRAS*, and *BRAF* mutations were investigated in archival formalin-fixed, paraffin-embedded tissue blocks or material from primary or metastatic lesions obtained from diagnostic and/or therapeutic procedures. All histologies were centrally reviewed at MD Anderson. Mutation testing was performed in the Clinical Laboratory Improvement Amendment–certified Molecular Diagnostic Laboratory within the Division of Pathology and Laboratory Medicine at MD Anderson. DNA was extracted from microdissected paraffin-embedded tumor sections and analyzed using a polymerase chain reaction-based DNA sequencing method for *PIK3CA* mutations in codons 532-554 of exon 9 (helical domain) and codons 1011-1062 of exon 20 (kinase domain). Analysis included the mutation hot spot region of the *PIK3CA* proto-oncogene denoted by Sanger sequencing, following amplification of 276 bp and 198 bp amplicons, respectively, utilizing primers designed by the MD Anderson Molecular Diagnostic Laboratory. Whenever possible, in addition to *PIK3CA*, mutation analysis was done for *KRAS* and *NRAS* codons 12, 13, and 61 mutations, and *BRAF* codons 595-600 mutations of exon 15 by pyrosequencing, as previously described.[19]

### Treatment

Prior to being treated with Phase I agents, patients typically received the US Food and Drug Administration (FDA)-approved therapy. If available, we collected data from the last FDA-approved therapy in addition to the Phase I therapy given in the CCTT. For patients with *PIK3CA* mutations, data were recorded for treatment received that included PI3K/AKT/mTOR inhibitors. For wt *PIK3CA* patients, data from the best phase I therapy were recorded. The response outcome was measured by partial [PR] or complete response [CR] or the absence of PR/CR as well as the duration of progression-free survival [PFS] and overall survival (OS). [20]

### Statistical analysis

Patient characteristics were summarized using descriptive statistics. Response to treatment (PR or CR) was evaluated using Response Evaluation Criteria in Solid Tumors (RECIST 1.0).[20] PFS was defined as the interval from initiation of the selected phase I treatment to disease progression or death. Patients who were alive and not progressing were censored at the date of the last follow-up. Overall survival (OS) was defined as the interval from diagnosis to death and OS on phase I therapy (OS-Ph1) was defined as the interval between recorded initiation of the systemic phase I treatment to death. Patients who were alive were censored at the date of the last follow-up. Distant metastasis-free survival (DMFS) was defined as the interval from diagnosis to development of metastatic disease. The probabilities of PFS, OS, OS-Ph1, and DMFS were estimated using the method of Kaplan and Meier and the time-to-event endpoints were compared among subgroups using the log-rank test.[21, 22]

Associations between *PIK3CA* mutation status and categorical variables (ethnicity, biopsy/tissue site, metastatic site, history of deep vein thrombosis [DVT], history of smoking, *KRAS* mutation, *NRAS* mutation, *BRAF* mutation, PR/CR status after last FDA-approved therapy, PR/CR status after PI3K/AKT/mTOR targeted phase I therapy in *PIK3CA*-mutant patients, or status after the best phase I therapy in wt *PIK3CA* patients) were assessed using Fisher's exact test. PR/CR rate to prior vs. current therapies in matched paired subjects was assessed using McNemar's test. In addition, univariate and multivariate logistic regression models were fit to assess the associations between *PIK3CA* mutations and other categorical variables. A Cox regression model was applied to assess the effect of covariates on time-to-event endpoints. All tests were two-sided, and a P value less than 0.05 was considered statistically significant. All statistical analyses were carried out using SPSS 17 computer software (SPSS Chicago, IL) and R version 2.15.0 (R Foundation for Statistical Computing).

## RESULTS

### Patient characteristics

A total of 270 patients with diverse advanced cancers consisting of 90 patients with *PIK3CA* mutations and 180 controls with wt *PIK3CA* (matched by tumor type, gender, and age) were analyzed and their clinical and pathologic characteristics are listed in Table 1. Most patients (79%) were white and women (62%). The median age was 56 years (range, 16-83) and patients received the median number of 3 prior therapies (range, 0-12). The most prevalent tumor types were colorectal cancer (27%), breast cancer (17%), ovarian cancer (12%) and endometrial cancer (10%). Lung and/or liver metastases were found in 62% of patients. Brain metastases were found in 13% of patients. Of the 208 patients tested for *KRAS*, 53 (25%) had a mutation; of the 95 tested for *NRAS*, 3 (3%) had a mutation; and of the 179 tested for *BRAF*, 15 (8%) had a mutation. When analyzing tested MAPK mutations, of the 132 patients tested for *KRAS*, *NRAS*, and *BRAF* mutation status (patients were selected for analyses if they had a mutation in *KRAS*, or *NRAS*, or *BRAF* since they are known to be mutually exclusive or if they were tested negative for all 3 oncogenes), 71 (54%) had *KRAS*, *NRAS* or *BRAF* mutations.

**Table 1:** Patient characteristics

Variable	Number (%)	*PIK3CA* mutation (%)	wild-type *PIK3CA* (%)	P value
All	270 (100)	90 (100)	180 (100)	Not applicable
Gender				
Man	102 (38)	34 (38)	68 (38)	1.00‡
Women	168 (62)	56 (62)	112 (62)	
Age				
</= 50 years	80 (30)	26 (29)	54 (30)	0.89‡
>50 years	190 (70)	64 (71)	126 (70)	
Ethnicity				
Caucasian	213 (79)	67 (74)	146 (81)	0.12
African-American	25 (9)	13 (14)	12 (7)	
Hispanic	18 (7)	4 (5)	14 (8)	
Asian	14 (5)	6 (7)	8 (4)	
Smoking History				
Past and current smokers	113 (42)	35 (39)	78 (43)	0.52
Non-smokers	157 (58)	55 (61)	102 (57)	
History of deep vein thrombosis				
Yes	53 (20)	24 (27)	29 (16)	0.05
No	217 (80)	66 (73)	151 (84)	
Prior therapies				
</= 3	143 (53)	52 (58)	91 (51)	0.30
>3	127 (47)	38 (42)	89 (49)	
Site of Primary Tumor				
Colorectal	72 (27)	24 (27)	48 (27)	1.00‡
Breast	45 (17)	15 (17)	30 (17)	
Ovarian	33 (12)	11 (12)	22 (12)	
Endometrial	27 (10)	9 (10)	18 (10)	
Head & neck: squamous	24 (9)	8 (9)	16 (9)	
Cervical: squamous	18 (7)	6 (7)	12 (7)	
Non-small cell lung	12 (4)	4 (4)	8 (4)	
Other	39 (14)	13 (14)	26 (14)	
Metastases				
Lungs	168 (62)	59 (66)	109 (61)	0.51
Liver	168 (62)	55 (61)	113 (63)	0.79
Brain	34 (13)	10 (11)	24 (13)	0.70
Bones	89 (33)	25 (28)	64 (36)	0.22
Mutations				
*KRAS* mutated*	53 (25)	25 (34)	28 (21)	0.047
*KRAS* wild-type*	155 (75)	49 (66)	106 (79)	
*NRAS* mutated**	3 (3)	1 (3)	2 (3)	1.00
*NRAS* wild-type**	92 (97)	31 (97)	61 (97)	
*BRAF* mutated¶	15 (8)	7 (11)	8 (7)	0.41
*BRAF* wild-type¶	164 (92)	58 (89)	106 (93)	
*RAS* (*K*- or *N*-) or *BRAF* mutated¶¶	71 (54)	33 (66)	38 (46)	0.03
*RAS* (*K*- or *N*-) and *BRAF* wild-type¶¶	61 (46)	17 (34)	44 (54)	

‡ Patients with *PIK3CA* mutations and controls with wild-type *PIK3CA* were matched by tumor type, gender, and age (+/− 5 years). Therefore, differences cannot be expected

* Tested for *KRAS*, n=208 (*PIK3CA* mutation, n=74; wild-type *PIK3CA*, n=134)

** Tested for *NRAS*, n=95 (*PIK3CA* mutation, n=32; wild-type *PIK3CA*, n=63)

¶ Tested for *BRAF*, n=179 (*PIK3CA* mutation, n=65; wild-type *PIK3CA*, n=114)

¶¶ Tested for *RAS* (K- or N-) or *BRAF*, n=132 (*PIK3CA* mutation, n=50; wild-type *PIK3CA*, n=82). Since mutations in *KRAS*, *NRAS*, *BRAF* are considered to be mutually exclusive, patients with mutations or patients tested negative for all three mutations were included in the analysis

### Mutation types

Of the 90 patients with *PIK3CA* mutations, 56 (62%) had mutations in exon 9 coding for the helical domain, 33 (37%) in exon 20 coding for the kinase domain, and 1 (1%) had a dual mutation in exons 9 and 20. The most frequent mutation types were E545K (1633G>A) in 31 (34%) patients, followed by mutated H1047R (3140A>G) in 20 (22%) patients and E542K (1624G>A) mutations in 16 (18%) patients (Table 2).

**Table 2:** Types of *PIK3CA*, *KRAS*, *NRAS* and *BRAF* mutations

Mutation type	N (%)
*PIK3CA* mutation	90 (100)
E542K	16 (18)
E542V	1 (<3)
E545K	31 (34)
E545G	2 (<3)
Q546K	2 (<3)
S553N	1 (<3)
P539R, E545A	1 (<3)
E545K, D549H	1 (<3)
Exon 9 deletion	1 (<3)
E545A, H1047Y	1 (<3)
R1023Q	1 (<3)
M1043I	2 (<3)
M1043V	2 (<3)
D1045N	1 (<3)
H1047L	4 (4)
H1047R	20 (22)
G1049R	3 (3)
KRAS* mutation	53 (100)
G12A	7 (13)
G12C	4 (8)
G12D	14 (26)
G12F	1 (<3)
G12R	3 (6)
G12S	2 (4)
G12V	10 (19)
G13D	5 (9)
Q61H	2 (4)
Q61L	1 (<3)
Not specified	4 (8)
NRAS** mutations	3 (100)
G13D	1 (33)
Q61K	1 (33)
Q61R	1 (33)
*BRAF*¶	15 (100)
V600E	11 (73)
V600K	3 (20)
V600R	1 (7)

* Tested for *KRAS*, n=208

** Tested for *NRAS*, n=95

¶ Tested for *BRAF*, n=179

Of the 53 patients with *KRAS* mutations, 14 (26%) had a G12D mutation (35G>A), 10 (19%) a G12V mutation (35G>T), 7 (13%) had a G12A mutation (35G>C), 5 (9%) had a G13D mutation (38G>A) and 17 (32%) had other mutations (Table 2).

Of the 3 patients with *NRAS* mutations, 1 (33%) had a G13D mutation (38G>A), 1 (33%) had a Q61K mutation (181C>A) and 1 (33%) had a Q61R mutation (182A>G) (Table 2).

Of the 15 patients with *BRAF* mutations, 11 (73%) had a V600E mutation (1799_1800TG>AA), 3 (20%) a V600K mutation (1798_1799GT>AA), and 1 (7%) a V600R mutation (1798_1799GT>AG) (Table 2).

For *KRAS*, *NRAS*, and *BRAF* mutations, no patient had more than one type of mutation within each gene, which is not surprising since these mutations are known to be mutually exclusive.[13, 23]

### Clinical and molecular features associated with PIK3CA mutations

Patients with *PIK3CA* mutations (n=90) compared to patients with wt *PIK3CA* (n=180) had a trend to a higher incidence of DVT (24/90 [27%] vs. 29/180 [16%], p=0.05), higher prevalence of *KRAS* mutations (25/74 [34%] vs. 28/134 [21%], p=0.047), and a higher prevalence of mutations in the MAPK pathway (*KRAS*, *NRAS*, or *BRAF* mutations) (33/50 [66%] vs. 38/82 [46%], p=0.03) (Table 1). There was no difference with respect to ethnicity, smoking history, number of prior therapies, biopsy/tissue site, and the occurrence of lung, liver, bone, and brain metastases between patients with and without *PIK3CA* mutations (Table 1). The multivariate regression model, which included MAPK mutation status and history of DVT, confirmed that patients with *PIK3CA* mutations had a higher prevalence of MAPK mutations (odds ratio [OR] 2.19, 95% confidence interval [CI] 1.05-4.59, p=0.04).

Disease-specific subanalyses showed a trend toward an association between *PIK3CA* and *KRAS* mutations in colorectal cancer (17/24 [71%] vs. 21/44 [48%]; p=0.08) and associations between *PIK3CA* mutations and *KRAS* mutations (6/17 [35%] vs. 2/28 [7%], p=0.04) and MAPK mutations (8/13 [62%] vs. 2/15 [13%], p=0.02) in ovarian and endometrial cancers combined.

In addition, in all tumor types we analyzed clinical and molecular associations separately for *PIK3CA* mutations in exon 9 (helical domain) and exon 20 (kinase domain), and found that *PIK3CA* mutations in exon 9 compared to others (wt *PIK3CA*, *PIK3CA* exon 20 mutations) had a trend toward an association with simultaneous *KRAS* mutations (17/46 [37%] vs. 36/162 [22%]; p=0.05), had a trend toward association with *BRAF* mutations (6/42 [14%] vs. 9/137 [7%]; p=0.12), and was significantly associated with MAPK mutations (23/33 [70%] vs. 48/99 [48%]; p=0.04). *PIK3CA* mutations in exon 20 compared to others (wt *PIK3CA*, *PIK3CA* exon 9 mutations) were not associated with *KRAS* mutations (8/28 [29%] vs. 45/180 [25%]; p=0.65), *BRAF* mutations (1/23 [7%] vs. 14/156 [13%]; p=0.70), or MAPK mutations (10/17 [59%] vs. 61/115 [53%]; p=0.80). In addition, *PIK3CA* mutations in exon 9 compared to others demonstrated a trend toward an association with a history of DVT (16/56 [29%] vs. 37/214 [17%]; p=0.09). There were no other associations with any other assessed characteristics (ethnicity, smoking history, number of prior therapies, biopsy/tissue site, and the occurrence of lung, liver, bone, and brain metastases).

### Treatment outcomes with respect to PIK3CA mutation status

We analyzed PR/CR rates from the last FDA-approved treatment in 197 patients with available data and found no statistically significant differences between patients with *PIK3CA* mutations and wt *PIK3CA* patients (5/59 [8%] vs. 6/138 [4%], p=0.31) (Table 3). In contrast, in 208 patients who received phase I systemic therapy, those with *PIK3CA* mutations treated with a phase I therapy targeting the PI3K/AKT/mTOR pathway had a higher PR/CR rate than wt *PIK3CA* patients treated with their best phase I therapy (10/56 [18%] vs. 12/152 [8%], p=0.045). We also analyzed PR/CR rate following phase I therapy separately for patients with exon 9 and those with exon 20 *PIK3CA* mutations. Patients with *PIK3CA* exon 9 mutations showed a trend toward a higher PR/CR rate to the phase I therapy with a PI3K/AKT/mTOR inhibitor than patients with wt *PIK3CA* treated with their best phase I therapy (4/30 [13%] vs. 6/138 [4%], p=0.08). Patients with *PIK3CA* exon 20 mutations had a higher PR/CR rate after phase I therapy with a PI3K/AKT/mTOR inhibitor compared to patients with wt *PIK3CA* treated with their best phase I therapy (6/25 [24%] vs. 6/138 [4%], p=0.004). In addition, we analyzed PR/CR rate from the phase I therapy separately for patients with the most frequent mutations: E545K (n=31), H1047R (n=20), and E542K (n=16). While there was no difference in PR/CR rates in patients with E545K or E542K mutations, patients with H1047R treated with a PI3K/AKT/mTOR inhibitor compared to wt *PIK3CA* treated with their best phase I therapy demonstrated higher PR/CR rates(6/16 [38%] vs. 12/152 [8%]; p=0.003).

**Table 3:** Outcomes in patients with *PIK3CA* mutations and wild-type *PIK3CA* patients

Cancer	Outcome	*PIK3CA* mutation	wild-type *PIK3CA*	OR or HR (95% CI)	P value
All	PR/CR last FDA	5/59 (8%)	6/138 (4%)	OR 2.04 (95% CI 0.60-6.96)	0.31
	PR/CR Phase I	10/56 (18%)	12/152 (8%)	OR 2.74 (95% CI 1.07.-7.01)	0.045
	PFS on last FDA (95% CI)	3.0 months (95% CI 2.6-3.4)	3.2 months (95% CI 2.5-3.9)	HR 1.01 (95% CI 0.80-1.50)	0.55
	PFS on Phase I (95% CI)	2.0 months (95% CI 1.4-2.6)	3.7 months (95% CI 3.2-4.2)	HR 1.10 (95% CI 0.78-1.56)	0.59
	DMFS (95% CI)	12.3 months (95% CI 7.5-17.1)	18.8 months (95% CI 14.5-23.1)	HR 1.08 (95% CI 0.77-1.53)	0.64
	OS (95% CI)	50.4 months (95% CI 36.2-64.6)	55.2 months (95% CI 46.7-63.7)	HR 1.07 (95% CI 0.77-1.47)	0.70
	OS-Ph1 (95% CI)	6.6 months (95% CI 3.9-9.3)	8.6 months (95% CI 7.1-10.1)	HR 1.49 (95% CI 1.04-2.14)	0.03
Colorectal	PR/CR last FDA (n=55)	1/18 (6%)	0/37 (0%)	NA	0.34
	PR/CR Phase I (n=47)	0/14 (0%)	0/33 (0%)	NA	NA
	PFS on last FDA	2.8 months (95% CI 1.6-4.0)	4.3 months (95% CI 3.3-5.3)	HR 1.62 (95% CI 0.90-2.92)	0.10
	PFS on Phase I	1.8 months (95% CI 1.5-2.1)	3.8 months (95% CI 3.5-4.1)	HR 1.86 (95% CI 0.93-3.73)	0.07
	DMFS	15.2 months (95% CI 5.3-25.1)	18.8 months (95% CI 4.4-33.2)	HR 0.99 (95% CI 0.43-2.29)	1.00
	OS	45.1 months (95% CI 36.3-53.9)	54.0 months (95% CI 33.2-74.8)	HR 1.13 (95% CI 0.62-2.06)	0.70
	OS-Ph1	3.6 months (95% CI 2.5-4.7)	10.3 months (95% CI 6.6-14.0)	HR 3.05 (95% CI 1.51-6.18)	0.001

Abbreviations: CI, confidence interval; FDA, Food and Drug Administration; HR, hazard ratio; NA, not applicable; OR, odds ratio; PR/CR, partial or complete response; PFS, progression-free survival; DMFS, distant metastases-free survival; OS-Ph1, overall survival on phase I therapy; OS, overall survival from diagnosis

We next analyzed PFS after the last FDA-approved therapy (n=197) and phase I systemic therapy (n=208) (Table 3). There was no significant difference in median PFS following the last FDA-approved therapy between patients with *PIK3CA* mutations (3 months, 95% CI 2.6-3.4) and wt *PIK3CA* (3.2 months, 95%CI 2.5-3.9, p=0.55). Similarly, there was no significant difference in median PFS after treatment with a phase I therapy targeting the PI3K/AKT/mTOR pathway in *PIK3CA*-mutant patients (2 months, 95% CI 1.4-2.6) versus treatment with the best systemic phase I therapy in wt *PIK3CA* patients (3.7 months, 95%CI 3.2-4.2, p=0.59). We also analyzed PFS from the phase I therapy separately for patients with exon 9 and exon 20 *PIK3CA* mutations. Patients with *PIK3CA* exon 9 mutations did not have a significantly different median PFS on phase I therapy compared to patients with wt *PIK3CA* (2 months [95%CI 1.9-2.1] vs. 3.7 months [95%CI 3.2-4.2], p=0.41). Similarly, patients with *PIK3CA* exon 20 mutations had no significantly different median PFS after phase I therapy compared to patients with wt *PIK3CA* (1.9 months [95%CI 0.8-3.0] vs. 3.7 months [95%CI 3.3-4.1], p=0.77). Patients with H1047R mutations compared to wt *PIK3CA* did not have a significantly different median PFS (5.7 months [95%CI 0.9-10.5] vs. 3.7 months [95%CI 3.2-4.2], p=0.26).

Next, we performed paired analysis in 143 patients who had available data for treatment with the last FDA-approved therapy and who then received subsequent phase I systemic therapy to compare PR/CR rate and PFS in these subgroups. Patients with *PIK3CA* mutations had a similar PR/CR rate in response to treatment with phase I therapies targeting the PI3K/AKT/mTOR pathway than to their previous FDA-approved therapy (5/36 [14%] vs. 2/36 [6%], p=0.38) (Table 4). There was no statistically significant difference in a median PFS on the last FDA-approved therapy (3.6 months, 95% CI 2.8-4.4) or phase I therapy targeting PI3K/AKT/mTOR (2.8 months, 95%CI 1.3-4.3) in patients with *PIK3CA* mutations (p=0.60). Patients with wt *PIK3CA* had a similar PR/CR rate to the best phase I and the last FDA-approved therapies (6/107 [6%] vs. 7/107 [7%], p=1.00). There was no statistically significant difference in median PFS after the last FDA-approved therapy (3.5 months, 95% CI 2.7-4.3) and after the best phase I therapy (3.6 months, 95%CI 3.1-4.4.1) in patients with *PIK3CA* mutations (p=0.37).

**Table 4:** Paired analysis of treatment outcomes on last FDA-approved and phase I therapy

Patients	Outcome	FDA approved	Phase I	P value
All *PIK3CA* mutations	PR/CR	2/36 (6%)	5/36 (14%)	0.38
	PFS	3.6 months (95% CI 2.8-4.4)	2.8 months (95% CI 1.3-4.3)	0.60
All wt *PIK3CA*	PR/CR	6/107 (6%)	7/107 (7%)	1.00
	PFS	3.5 months (95% CI 2.7-4.3)	3.6 months (95% CI 3.1-4.1)	0.37
Colorectal *PIK3CA* mutations	PR/CR	0/10 (0%)	0/10 (0%)	1.00
	PFS	3.7 months (95% CI 2.0-5.4)	1.7 months (95% CI 1.2-2.2)	0.01
Colorectal wt *PIK3CA*	PR/CR	0/25 (0%)	0/25 (0%)	1.00
	PFS	5.0 months (95% CI 4.1-5.9)	3.9 months (95% CI 3.2-4.6)	0.34

Abbreviations: CI, confidence interval; FDA, Food and Drug Administration; PR/CR, partial or complete response; PFS, progression-free survival; wt, wild-type

Finally we analyzed OS (measured from diagnosis), OS-Ph1 (measured from recorded phase I therapy), and DMFS (measured from diagnosis to metastatic disease) for patients with *PIK3CA* mutations and wt *PIK3CA* patients (Table 3). Patients with *PIK3CA* mutations had a similar median OS (50.4 months, 95% CI 36.2-64.6) as patients with wt *PIK3CA* (55.2 months, 95% CI 46.7-63.7, p=0.70). In 142 patients who were initially diagnosed with localized disease, those with *PIK3CA* mutations had a numerically shorter DMFS (12.3 months, 95% CI 7.5-17.1) than patients with wt *PIK3CA* (18.8 months, 95% CI 14.5-23.1, p=0.6). In 208 patients who received phase I therapy, those with *PIK3CA* mutations had a shorter median Ph1-OS (6.6 months, 95% CI 3.9-9.3) than patients with wt *PIK3CA* (8.6 months, 95% CI 7.1-10.1, p=0.03).

Colorectal cancer was the largest tumor-specific subgroup, consisting of 24 patients with *PIK3CA* mutations and 48 matched wt *PIK3CA* controls. We therefore performed subanalysis on this histology. *PIK3CA* mutations were most frequent in exon 9 (16/24 [67%]) (Supplementary Table 1). *PIK3CA* mutations were not significantly associated with any specific clinical characteristics, although there was a trend to a lower prevalence of liver metastases compared to patients with wt *PIK3CA* (16/24 [67%] vs. 40/48 [83%], p=0.14) (Supplementary Table 2). In patients tested for *KRAS* mutations, those with *PIK3CA* mutations compared to wt *PIK3CA* demonstrated a trend to having a higher prevalence of *KRAS* mutations (17/24 [71%] vs. 21/44 [48%]; p=0.08) (Supplementary Table 2). In a separate analysis, *PIK3CA* exon 9 mutations compared to others had a trend to a higher frequency of *KRAS* mutations (12/16 [75%] vs. 26/52 [50%]; p=0.09), whereas *PIK3CA* exon 20 mutations compared to others showed no association with *KRAS* mutations (5/8 [63%] vs. 33/60 [55%]; p=1.00).

There was no significant difference between colorectal cancer patients with *PIK3CA* mutations and wt *PIK3CA* in PR/CR rate to the last line of FDA-approved therapy (1/18 [6%] vs. 0/37 [0%]; p=0.34) (Table 3). Patients with *PIK3CA* mutations had no response to PI3K/AKT/mTOR-targeted phase I therapies and, similarly, wt *PIK3CA* patients did not respond to the best phase I therapy (0/14 [0%] vs. 0/33 [0%]; p=1.00).

Colorectal cancer patients with *PIK3CA* mutations compared to wt *PIK3CA* demonstrated a trend to a shorter median PFS to the last FDA-approved therapy (2.8 months [1.6-4.0] vs. 4.3 months [3.3-5.3]; p=0.10) (Table 3). Similarly, patients with *PIK3CA* mutations treated with a PI3K/AKT/mTOR targeted therapy compared to wt *PIK3CA* patients treated with their best phase I therapy showed a trend to a shorter median PFS (1.8 months [1.5-2.1] vs. 3.8 months [3.5-4.1]; p=0.07).

A paired analysis of colorectal cancer patients for whom we had data on the last FDA-approved therapy and phase I therapy (*PIK3CA* mutations, n=10; wt *PIK3CA*, n=25), there was no response noted (Table 4). *PIK3CA* mutant patients had a significantly longer PFS on the last FDA-approved therapy compared to phase I PI3K/AKT/mTOR targeted therapy (3.7 months [2.0-5.4] vs. 1.7 months [1.2-2.2]; p=0.01). In wt *PIK3CA* patients, there was no significant difference in median PFS on the last FDA-approved therapy compared to best phase I therapy (5 months [4.1-5.9] vs. 3.9 months [3.2-4.6], p=0.34).

Finally, there was no significant difference in OS, OS-Ph1, and DMFS between colorectal cancer patients with *PIK3CA* mutations and wt *PIK3CA* (Table 3).

## DISCUSSION

In this study of 90 patients with *PIK3CA* mutations and 180 wt *PIK3CA* controls (matched by tumor type, gender, and age) we identified having a history of DVT as the only clinical characteristic potentially associated with *PIK3CA* mutations. However, this association was not confirmed by multivariate analysis. None of the clinical characteristics including ethnicity, the site of metastases, smoking history, number of prior therapies, OS, OS-Ph1, DMFS was associated with *PIK3CA* status.

In agreement with previous reports, when assessing individuals for MAPK mutations we noted the association between *PIK3CA* and MAPK mutations (66% vs. 46%, p=0.03) and between *PIK3CA* and *KRAS* mutations (34% vs. 21%, p=0.047).[13, 14] In disease-specific sub-analyses, also in agreement with previous reports, there was a trend toward an association between *PIK3CA* and *KRAS* mutations in colorectal cancer (71% vs. 48%, p=0.08); associations between *PIK3CA* and *KRAS* (35% vs. 7%, p=0.04), and *PIK3CA* and MAPK mutations (62% vs. 13%, p=0.02) in ovarian and endometrial cancers.[13, 14] We also looked for associations linked to mutations in exon 9 and exon 20, which account for more than 80% of *PIK3CA* mutations.[24] Overall, *PIK3CA* exon 9 mutations were associated with simultaneous MAPK mutations (70% vs. 48%; p=0.04), showed a trend toward association with simultaneous *KRAS* mutations (37% vs. 22%; p=0.05), and albeit a weaker trend toward association with *BRAF* mutations (14% vs. 7%; p=0.12), whereas *PIK3CA* exon 20 mutations were not associated with any mutations in the MAPK pathway. This finding is consistent with previous reports from our group and others in colorectal cancer and other tumor histologies.[13, 14]

Oncogenic mutations often point to the presence of a therapeutic target that might be amenable to directed therapeutic intervention. For example, *KIT* mutations render patients with gastrointestinal stromal tumor sensitive to KIT tyrosine kinase inhibitors (TKIs), *EGFR* mutations render patients with NSCLC sensitive to EGFR TKIs, a *EML4-ALK* fusion renders patients with NSCLC sensitive to ALK inhibitors, and *BRAF* mutations increase the sensitivity of melanoma patients to BRAF inhibitors.[25-28] We reported increased PR/CR rates in response to PI3K/AKT/mTOR inhibitors in patients with *PIK3CA* mutations compared to wt *PIK3CA* treated in early phase clinical trials.[8] In the current study, we retrospectively evaluated treatment outcomes (PR/CR rate, PFS) on the last FDA-approved therapy and phase I therapy (therapy targeting PI3K/AKT/mTOR pathway in patients with *PIK3CA* mutations or best phase I therapy [defined by CR/PR or longest PFS] in patients with wt *PIK3CA*). Overall, there was no difference in PR/CR rate (8% vs. 4%; p=0.31) and PFS (3.0 months vs. 3.2 months, p=0.55) to the last FDA-approved therapy between patients with *PIK3CA* mutations and wt *PIK3CA*, but patients with *PIK3CA* mutations had a higher PR/CR rate to phase I therapy with PI3K/AKT/mTOR inhibitors (18% vs. 8%; p=0.045), which did not, however, translate to a longer PFS (2.0 months vs. 3.7 months; p=0.59) compared to wt *PIK3CA* patients treated with their best phase I therapy. In the paired analysis, which included only patients who had available data from treatment with both the last FDA-approved therapy and phase I therapy, we found no difference in PR/CR rates and PFS in patients with *PIK3CA* mutations and wt *PIK3CA*. The explanation for this might be because PI3K/AKT/mTOR targeting therapies are effective in only a subset of patients. In NSCLC, specific *EGFR* mutations such as exon 19 deletions or exon 21 mutations (L858R), render tumors sensitive to EGFR TKIs, whereas an exon 20 mutation (T790M) is associated with therapeutic resistance.[29] A similar scenario is possible for other oncogenic mutations. Indeed, in our current study we noticed that patients with H1047R *PIK3CA* mutations compared to patients with wt *PIK3CA* had an increased PR/CR rate (38% vs. 8%; p=0.003) to phase I therapy compared to wt *PIK3CA* patients. Another explanation might relate to the histological milieu of the *PIK3CA* mutations. Although, these mutations were found in a variety of cancers, the largest subgroup of patients had colorectal cancer. These patients showed a poor outcome on PI3K/AKT/mTOR, perhaps because of the frequent coexistence of *KRAS* mutations in this histology.[13, 14, 30]

Finally, we investigated whether *PIK3CA* mutations have prognostic significance. For instance, *EGFR* mutations compared to wt *EGFR* in NSCLC are usually associated with improved treatment outcomes and a longer OS.[31] *KRAS* mutations in NSCLC are associated with poor outcomes in response to EGFR therapies.[31] In colorectal cancer, BRAF mutations are associated with a shorter survival.[13] Consensus regarding the impact of *PIK3CA* mutations is contradictory. Some investigators reported better prognosis in certain cancers such as breast cancer with *PIK3CA* mutations, whereas others suggested that *PIK3CA* mutations indicate a worse prognosis in colorectal cancer, endometrial cancer and NSCLC.[13, 29, 32-36] There was no significant difference in patients with *PIK3CA* mutations and wt *PIK3CA* in OS (from the time of first diagnosis; 50.4 months vs. 55.2 months; p=0.70), and DMFS (from time of first diagnosis to development of metastatic disease; 12.3 months vs. 18.8 months; p=0.64); however, patients with *PIK3CA* mutations had a shorter OS-Ph1 (from initiation of phase I therapy; 6.6 months vs. 8.6 months; p=0.03). In disease-specific sub-analyses, we found a lower survival in patients with colorectal cancer and *PIK3CA* mutations who, compared to wt *PIK3CA* patients, experienced a shorter OS-Ph1 (3.6 months vs. 10.3 months; p=0.001). This finding provides another piece of evidence suggesting that colorectal cancer patients with *PIK3CA* mutations do not do well on a PI3K/AKT/mTOR targeted therapy.

In conclusion, in the current study of 90 patients with *PIK3CA* mutations and 180 matched controls with wt *PIK3CA* we found that there was no *PIK3CA* phenotype. Overall, *PIK3CA* mutations were associated with *KRAS* and MAPK (*KRAS*, *NRAS*, *BRAF*) mutations. Patients with *PIK3CA* mutations treated with PI3K/AKT/mTOR axis inhibitors had a PR/CR rate of 18%. This PR/CR rate is lower than the rate we previously reported for gynecologic and breast malignancies, in which the PR/CR rate was 30%, and may be due to the fact that the largest subgroup (colorectal cancer) in this paper did not respond well to PI3K/AKT/mTOR axis therapy even in the presence of *PIK3CA* mutations.[16] The lack of response may be due to the high rate of concomitant MAPK mutations in colorectal cancer. Finally, patients with *PIK3CA* H1047R mutations in exon 20 appeared to have the most favorable PR/CR rate, but this observation requires confirmation in a larger cohort of patients.

## Supplementary Table



## References

[1] Engelman JA, Chen L, Tan X, Crosby K, Guimaraes AR, Upadhyay R, Maira M, McNamara K, Perera SA, Song Y, Chirieac LR, Kaur R, Lightbown A, Simendinger J, Li T, Padera RF (2008). Effective use of PI3K and MEK inhibitors to treat mutant Kras G12D and PIK3CA H1047R murine lung cancers. Nat Med.

[2] Yuan W, Stawiski E, Janakiraman V, Chan E, Durinck S, Edgar KA, Kljavin NM, Rivers CS, Gnad F, Roose-Girma M, Haverty PM, Fedorowicz G, Heldens S, Soriano RH, Zhang Z, Wallin JJ (2012). Conditional activation of Pik3ca(H1047R) in a knock-in mouse model promotes mammary tumorigenesis and emergence of mutations. Oncogene.

[3] Schwarz JK, Payton JE, Rashmi R, Xiang T, Jia Y, Huettner P, Rogers BE, Yang Q, Watson M, Rader JS, Grigsby PW (2012). Pathway-specific analysis of gene expression data identifies the PI3K/Akt pathway as a novel therapeutic target in cervical cancer. Clin Cancer Res.

[4] Miller TW, Balko JM, Arteaga CL (2011). Phosphatidylinositol 3-kinase and antiestrogen resistance in breast cancer. J Clin Oncol.

[5] Adams JR, Schachter NF, Liu JC, Zacksenhaus E, Egan SE (2011). Elevated PI3K signaling drives multiple breast cancer subtypes. Oncotarget.

[6] Ihle NT, Lemos R, Wipf P, Yacoub A, Mitchell C, Siwak D, Mills GB, Dent P, Kirkpatrick DL, Powis G (2009). Mutations in the phosphatidylinositol-3-kinase pathway predict for antitumor activity of the inhibitor PX-866 whereas oncogenic Ras is a dominant predictor for resistance. Cancer Res.

[7] Di Nicolantonio F, Arena S, Tabernero J, Grosso S, Molinari F, Macarulla T, Russo M, Cancelliere C, Zecchin D, Mazzucchelli L, Sasazuki T, Shirasawa S, Geuna M, Frattini M, Baselga J, Gallicchio M (2010). Deregulation of the PI3K and KRAS signaling pathways in human cancer cells determines their response to everolimus. J Clin Invest.

[8] Janku F, Tsimberidou AM, Garrido-Laguna I, Wang X, Luthra R, Hong DS, Naing A, Falchook GS, Moroney JW, Piha-Paul SA, Wheler JJ, Moulder SL, Fu S, Kurzrock R (2011). PIK3CA Mutations in Patients with Advanced Cancers Treated with PI3K/AKT/mTOR Axis Inhibitors. Mol Cancer Ther.

[9] Shimizu T, Tolcher AW, Papadopoulos KP, Beeram M, Rasco DW, Smith LS, Gunn S, Smetzer L, Mays TA, Kaiser B, Wick MJ, Alvarez C, Cavazos A, Mangold GL, Patnaik A (2012). The Clinical Effect of the Dual-Targeting Strategy Involving PI3K/AKT/mTOR and RAS/MEK/ERK Pathways in Patients with Advanced Cancer. Clin Cancer Res.

[10] Weigelt B, Warne PH, Downward J (2011). PIK3CA mutation, but not PTEN loss of function, determines the sensitivity of breast cancer cells to mTOR inhibitory drugs. Oncogene.

[11] Weber GL, Parat MO, Binder ZA, Gallia GL, Riggins GJ (2011). Abrogation of PIK3CA or PIK3R1 reduces proliferation, migration, and invasion in glioblastoma multiforme cells. Oncotarget.

[12] Garrett JT, Chakrabarty A, Arteaga CL (2011). Will PI3K pathway inhibitors be effective as single agents in patients with cancer?. Oncotarget.

[13] De Roock W, Claes B, Bernasconi D, De Schutter J, Biesmans B, Fountzilas G, Kalogeras KT, Kotoula V, Papamichael D, Laurent-Puig P, Penault-Llorca F, Rougier P, Vincenzi B, Santini D, Tonini G, Cappuzzo F (2010). Effects of KRAS, BRAF, NRAS, and PIK3CA mutations on the efficacy of cetuximab plus chemotherapy in chemotherapy-refractory metastatic colorectal cancer: a retrospective consortium analysis. Lancet Oncol.

[14] Janku F, Lee JJ, Tsimberidou AM, Hong DS, Naing A, Falchook GS, Fu S, Luthra R, Garrido-Laguna I, Kurzrock R (2011). PIK3CA mutations frequently coexist with RAS and BRAF mutations in patients with advanced cancers. PLoS One.

[15] Chaft JE, Arcila ME, Paik PK, Lau C, Riely GJ, Pietanza MC, Zakowski MF, Rusch V, Sima CS, Ladanyi M, Kris MG (2012). Coexistence of PIK3CA and other oncogene mutations in lung adenocarcinoma-rationale for comprehensive mutation profiling. Mol Cancer Ther.

[16] Janku F, Wheler JJ, Westin SN, Moulder SL, Naing A, Tsimberidou AM, Fu S, Falchook GS, Hong DS, Garrido-Laguna I, Luthra R, Lee JJ, Lu KH, Kurzrock R (2012). PI3K/AKT/mTOR inhibitors in patients with breast and gynecologic malignancies harboring PIK3CA mutations. J Clin Oncol.

[17] El-Osta H, Falchook G, Tsimberidou A, Hong D, Naing A, Kim K, Wen S, Janku F, Kurzrock R (2011). BRAF mutations in advanced cancers: clinical characteristics and outcomes. PLoS One.

[18] Tie J, Lipton L, Desai J, Gibbs P, Jorissen RN, Christie M, Drummond KJ, Thomson BN, Usatoff V, Evans PM, Pick AW, Knight S, Carne PW, Berry R, Polglase A, McMurrick P (2011). KRAS mutation is associated with lung metastasis in patients with curatively resected colorectal cancer. Clin Cancer Res.

[19] Zuo Z, Chen SS, Chandra PK, Galbincea JM, Soape M, Doan S, Barkoh BA, Koeppen H, Medeiros LJ, Luthra R (2009). Application of COLD-PCR for improved detection of KRAS mutations in clinical samples. Mod Pathol.

[20] Therasse P, Arbuck SG, Eisenhauer EA, Wanders J, Kaplan RS, Rubinstein L, Verweij J, Van Glabbeke M, van Oosterom AT, Christian MC, Gwyther SG (2000). New guidelines to evaluate the response to treatment in solid tumors. European Organization for Research and Treatment of Cancer, National Cancer Institute of the United States, National Cancer Institute of Canada. J Natl Cancer Inst.

[21] Kaplan EM (1958). P. Nonparametric estimator from incomplete observations. J American Statistical Association.

[22] Mantel N (1966). Evaluation of survival data and two new rank order statistics arising in its consideration. Cancer Chemother Rep.

[23] Goel VK, Lazar AJ, Warneke CL, Redston MS, Haluska FG (2006). Examination of mutations in BRAF, NRAS, and PTEN in primary cutaneous melanoma. J Invest Dermatol.

[24] Engelman JA (2009). Targeting PI3K signalling in cancer: opportunities, challenges and limitations. Nat Rev Cancer.

[25] van Oosterom AT, Judson I, Verweij J, Stroobants S, Donato di Paola E, Dimitrijevic S, Martens M, Webb A, Sciot R, Van Glabbeke M, Silberman S, Nielsen OS (2001). Safety and efficacy of imatinib (STI571) in metastatic gastrointestinal stromal tumours: a phase I study. Lancet.

[26] Maemondo M, Inoue A, Kobayashi K, Sugawara S, Oizumi S, Isobe H, Gemma A, Harada M, Yoshizawa H, Kinoshita I, Fujita Y, Okinaga S, Hirano H, Yoshimori K, Harada T, Ogura T (2010). Gefitinib or chemotherapy for non-small-cell lung cancer with mutated EGFR. N Engl J Med.

[27] Kwak EL, Bang YJ, Camidge DR, Shaw AT, Solomon B, Maki RG, Ou SH, Dezube BJ, Janne PA, Costa DB, Varella-Garcia M, Kim WH, Lynch TJ, Fidias P, Stubbs H, Engelman JA (2010). Anaplastic lymphoma kinase inhibition in non-small-cell lung cancer. N Engl J Med.

[28] Flaherty KT, Puzanov I, Kim KB, Ribas A, McArthur GA, Sosman JA, O'Dwyer PJ, Lee RJ, Grippo JF, Nolop K, Chapman PB (2010). Inhibition of mutated, activated BRAF in metastatic melanoma. N Engl J Med.

[29] Janku F, Garrido-Laguna I, Petruzelka LB, Stewart DJ, Kurzrock R, Janku F, Garrido-Laguna I, Petruzelka LB, Stewart DJ, Kurzrock R (2011). Novel therapeutic targets in non-small cell lung cancer. J Thorac Oncol.

[30] Chappell WH, Steelman LS, Long JM, Kempf RC, Abrams SL, Franklin RA, Basecke J, Stivala F, Donia M, Fagone P, Malaponte G, Mazzarino MC, Nicoletti F, Libra M, Maksimovic-Ivanic D, Mijatovic S (2011). Ras/Raf/MEK/ERK and PI3K/PTEN/Akt/mTOR inhibitors: rationale and importance to inhibiting these pathways in human health. Oncotarget.

[31] Douillard JY, Shepherd FA, Hirsh V, Mok T, Socinski MA, Gervais R, Liao ML, Bischoff H, Reck M, Sellers MV, Watkins CL, Speake G, Armour AA, Kim ES (2010). Molecular Predictors of Outcome With Gefitinib and Docetaxel in Previously Treated Non-Small-Cell Lung Cancer: Data From the Randomized Phase III INTEREST Trial. J Clin Oncol.

[32] Ludovini V, Bianconi F, Pistola L, Chiari R, Minotti V, Colella R, Giuffrida D, Tofanetti FR, Siggillino A, Flacco A, Baldelli E, Iacono D, Mameli MG, Cavaliere A, Crino L (2011). Phosphoinositide-3-kinase catalytic alpha and KRAS mutations are important predictors of resistance to therapy with epidermal growth factor receptor tyrosine kinase inhibitors in patients with advanced non-small cell lung cancer. J Thorac Oncol.

[33] He Y, Van't Veer LJ, Mikolajewska-Hanclich I, van Velthuysen ML, Zeestraten EC, Nagtegaal ID, van de Velde CJ, Marijnen CA (2009). PIK3CA mutations predict local recurrences in rectal cancer patients. Clin Cancer Res.

[34] Ogino S, Nosho K, Kirkner GJ, Shima K, Irahara N, Kure S, Chan AT, Engelman JA, Kraft P, Cantley LC, Giovannucci EL, Fuchs CS (2009). PIK3CA mutation is associated with poor prognosis among patients with curatively resected colon cancer. J Clin Oncol.

[35] Kalinsky K, Jacks LM, Heguy A, Patil S, Drobnjak M, Bhanot UK, Hedvat CV, Traina TA, Solit D, Gerald W, Moynahan ME (2009). PIK3CA mutation associates with improved outcome in breast cancer. Clin Cancer Res.

[36] Catasus L, Gallardo A, Cuatrecasas M, Prat J (2008). PIK3CA mutations in the kinase domain (exon 20) of uterine endometrial adenocarcinomas are associated with adverse prognostic parameters. Mod Pathol.

