# Supplementary material for: PIK3CA Mutations in Advanced Cancers: Characteristics and Outcomes
Source: Oncotarget. 2012 Nov 30;3(12):1566–75. doi: 10.18632/oncotarget.716 (PMC3681495; doi:10.18632/oncotarget.716)
Supplement: Supplementary file 1 [file oncotarget-03-1566-s001.doc]

PIK3CA Mutations in Advanced Cancers: Characteristics and Outcomes –

Janku et al

**Supplementary Table 1: Types of *PIK3CA*, *KRAS*, *NRAS* and *BRAF* mutations**

| Cancer | Domain/exon | Mutation | N (%) |
| --- | --- | --- | --- |
| Colorectal cancer (n=24) | Helical/ exon 9 | E542K | 6 |
|  | (n=16) | E545K | 10 |
|  | Kinase/exon 20 | H1047L | 3 |
|  | (n=8) | H1047R | 2 |
|  |  | other exon 20 | 3 |

**Supplementary Table 2: Clinical and molecular characteristics in patients with *PIK3CA* mutations and wild-type *PIK3CA*** and colorectal cancer

| Type of cancer | Variable | *PIK3CA* mutation (%) | wild-type *PIK3CA* (%) | P value |
| --- | --- | --- | --- | --- |
| Colorectal (n=72) | All | 24 | 48 |  |
|  | Lungs | 18 (75) | 39 (81) | 0.55 |
|  | Liver | 16 (67) | 40 (83) | 0.14 |
|  | Bones | 7 (29) | 13 (27) | 1.00 |
|  | Brain | 1 (4) | 0 (0) | 0.34 |
|  | DVT | 7 (29) | 12 (26) | 0.78 |
|  | *KRAS* mutation^*^ | 17 (71) | 21 (48) | 0.08 |
|  | MAPK mutations^**^ | 18 (95) | 27 (84) | 0.39 |

^*^ Tested for *KRAS*, n=68 (*PIK3CA* mutation, n=24; wild-type *PIK3CA*, n=44)

^**^ Tested for MAPK mutations, n=50 (*PIK3CA* mutation, n=19; wild-type *PIK3CA*, n=32)
